# The genome sequence of the Norway rat, *Rattus norvegicus* Berkenhout 1769

**DOI:** 10.12688/wellcomeopenres.16854.1

**Published:** 2021-05-18

**Authors:** Kerstin Howe, Melinda Dwinell, Mary Shimoyama, Craig Corton, Emma Betteridge, Alexander Dove, Michael A. Quail, Michelle Smith, Laura Saba, Robert W. Williams, Hao Chen, Anne E. Kwitek, Shane A. McCarthy, Marcela Uliano-Silva, William Chow, Alan Tracey, James Torrance, Ying Sims, Richard Challis, Jonathan Threlfall, Mark Blaxter

**Affiliations:** 1 Wellcome Sanger Institute, Wellcome Genome Campus, Hinxton, Cambridge, CB10 1SA, UK; 2 Medical College of Wisconsin, Milwaukee, Wisconsin, 53226, USA; 3 Skaggs School of Pharmacy and Pharmaceutical Sciences,, University of Colorado Anschutz Medical Center, Aurora, Colorado, 80045, USA; 4 Department of Genetics, Genomics and Informatics, University of Tennessee Health Science Center, Memphis, Tennessee, 38103, USA; 5 Department of Pharmacology, Addiction Science, and Toxicology, University of Tennessee Health Science Center, Memphis, Tennessee, 38103, USA; 6 Department of Genetics, University of Cambridge, Cambridge, CB2 3EH, UK

**Keywords:** Rattus norvegicus, Norway rat, genome sequence, chromosomal, reference genome

## Abstract

We present a genome assembly from an individual male *Rattus norvegicus* (the Norway rat; Chordata; Mammalia; Rodentia; Muridae). The genome sequence is 2.44 gigabases in span. The majority of the assembly is scaffolded into 20 chromosomal pseudomolecules, with both X and Y sex chromosomes assembled. This genome assembly, mRatBN7.2, represents the new reference genome for *R. norvegicus* and has been adopted by the Genome Reference Consortium.

## Species taxonomy

Eukaryota; Metazoa; Chordata; Mammalia; Rodentia; Muridae; Rattus; *Rattus norvegicus* Berkenhout 1769 (NCBI:txid10116).

## Introduction

*Rattus norvegicus* is one of the most well-established experimental model organisms, with use of the species dating back to the mid-19th century ( Modlinska & Pisula, 2020). The longstanding use of *R. norvegicus* in the laboratory as a model organism has led to a multitude of discoveries, providing insight into human physiology, behaviour and disease. The complexity of *R. norvegicus* relative to many other model organisms, in addition to its well-characterised physiology, means that it is frequently used in cancer research, behavioral neuroscience, and the pharmaceutical industry.

We present the reference genome mRatBN7.2 for the Norway rat, *Rattus norvegicus*. This genome assembly represents a substantial improvement on the previous assemblies, correcting areas of potential mis-assembly in the 2014 reference assembly, Rnor_6.0 ( Ramdas *et al.*, 2019). The new reference has a mean genome coverage of ^~^92x for a single male individual of the BN/NHsdMcwi strain, which was obtained from the same colony as the original “Eve” rat that was sampled 18 years ago for use in previous rat reference genome assemblies (Eve was a female rat of generation F14, the index male described here is generation F61). The new assembly contains no gaps between scaffolds and has a scaffold N50 an order of magnitude higher than the previous reference assembly; with just 756 contigs (N50 >29 Mb), its contiguity is comparable to that of reference assemblies for humans and mice.

The production of a high-quality reference genome assembly for *R. norvegicus* allows researchers using rats for research, as a model organism for human diseases, and for determining drug interactions to have as complete and reliable a genome as possible. The result is a greater depth and certainty in data interpretation and species comparison, which will have numerous benefits for biological understanding and health.

## Genome sequence report

**Figure 1.**
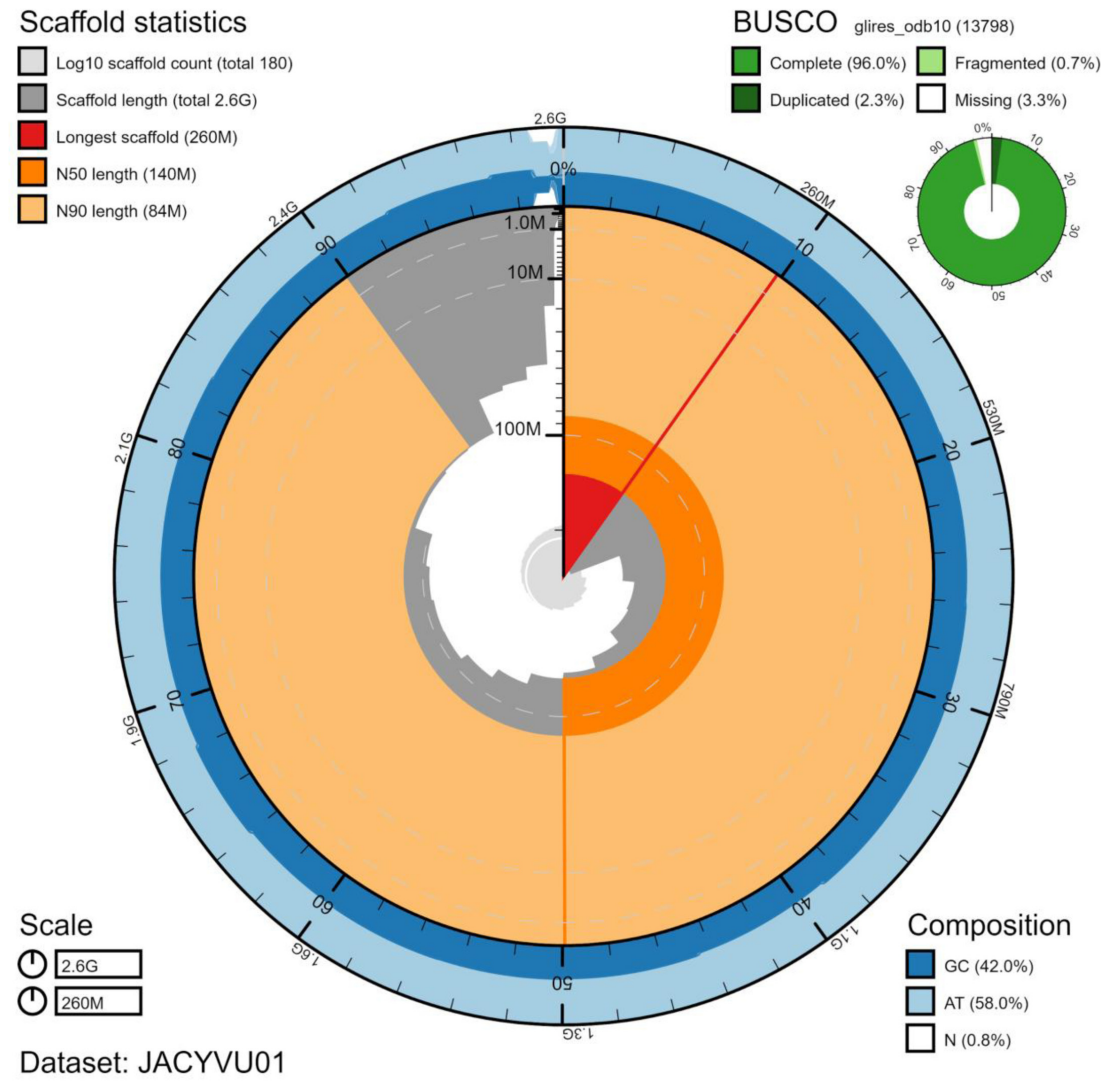
Genome assembly of *Rattus norvegicus*, mRatBN7.2: metrics. The BlobToolKit Snailplot shows N50 metrics and BUSCO gene completeness. An interactive version of this figure is available at https://blobtoolkit.genomehubs.org/view/Rattus%20norvegicus/dataset/JACYVU01/snail.

The genome was sequenced from the kidney tissue of a single male *R. norvegicus* (strain BN/NHsdMcwi, generation F61) housed at the Medical College of Wisconsin, Milwaukee, Wisconsin, USA. A total of 80-fold coverage in Pacific Biosciences single-molecule long reads (N50, 37 kb) and 31-fold coverage in 10X Genomics read clouds (from molecules with an estimated N50 of 26 kb) were generated. Primary assembly contigs were scaffolded with chromosome conformation Hi-C data (29-fold coverage). Manual assembly curation corrected 234 missing/misjoins and removed 34 haplotypic duplications, reducing the scaffold number by 4.8%, increasing the scaffold N50 by 0.04% and decreasing the assembly length by 0.9%. The final assembly has a total length of 2.65 Gb in 219 sequence scaffolds with a scaffold N50 of 135.0 Mb ( Table 1). The majority, 99.7%, of the assembly sequence was assigned to 20 chromosomal-level scaffolds representing 20 autosomes and the X and Y sex chromosomes ( Figure 1– Figure 4; Table 2). The assembly has a BUSCO ( Simão *et al.*, 2015) completeness of 96.2% using the mammalia_odb10 reference set. The primary assembly is a large-scale mosaic of both haplotypes (i.e. is not fully phased) and we have therefore also deposited the contigs corresponding to the alternate haplotype.

**Table 1.** Genome data for *R. norvegicus*.

*Project accession data*	
Assembly identifier	mRatBN7.2
Species	*Rattus norvegicus*
Specimen	mRatNor1
NCBI taxonomy ID	10116
BioProject	PRJNA662962
BioSample ID	SAMN16261960, SAMEA5928170
Isolate information	Laboratory animal, male, kidney tissue
*Raw data accessions*	
PacificBiosciences SEQUEL II	ERR5310326-ERR5310327
10X Genomics Illumina	ERR5309015-ERR5309022
Hi-C Illumina	ERR5309023, ERR5309024
BioNano	ERZ1741012
*Genome assembly*	
Assembly accession	GCA_015227675.2
Accession of alternate haplotype	GCA_015244455.1
Span (Mb)	2,648
Number of contigs	738
Contig N50 length (Mb)	34
Number of scaffolds	219
Scaffold N50 length (Mb)	135
Longest scaffold (Mb)	260
BUSCO * genome score	C:96.2%[S:94.0,D:2.2%],F:0. 9%,M:2.8%,n:9226

*BUSCO scores based on the mammalia_odb10 BUSCO set using v5.0.0. C= complete [S= single copy, D=duplicated], F=fragmented, M=missing, n=number of orthologues in comparison. A full set of BUSCO scores is available at https://blobtoolkit.genomehubs.org/view/Rattus%20norvegicus/dataset/JACYVU01/busco.

**Figure 2.**
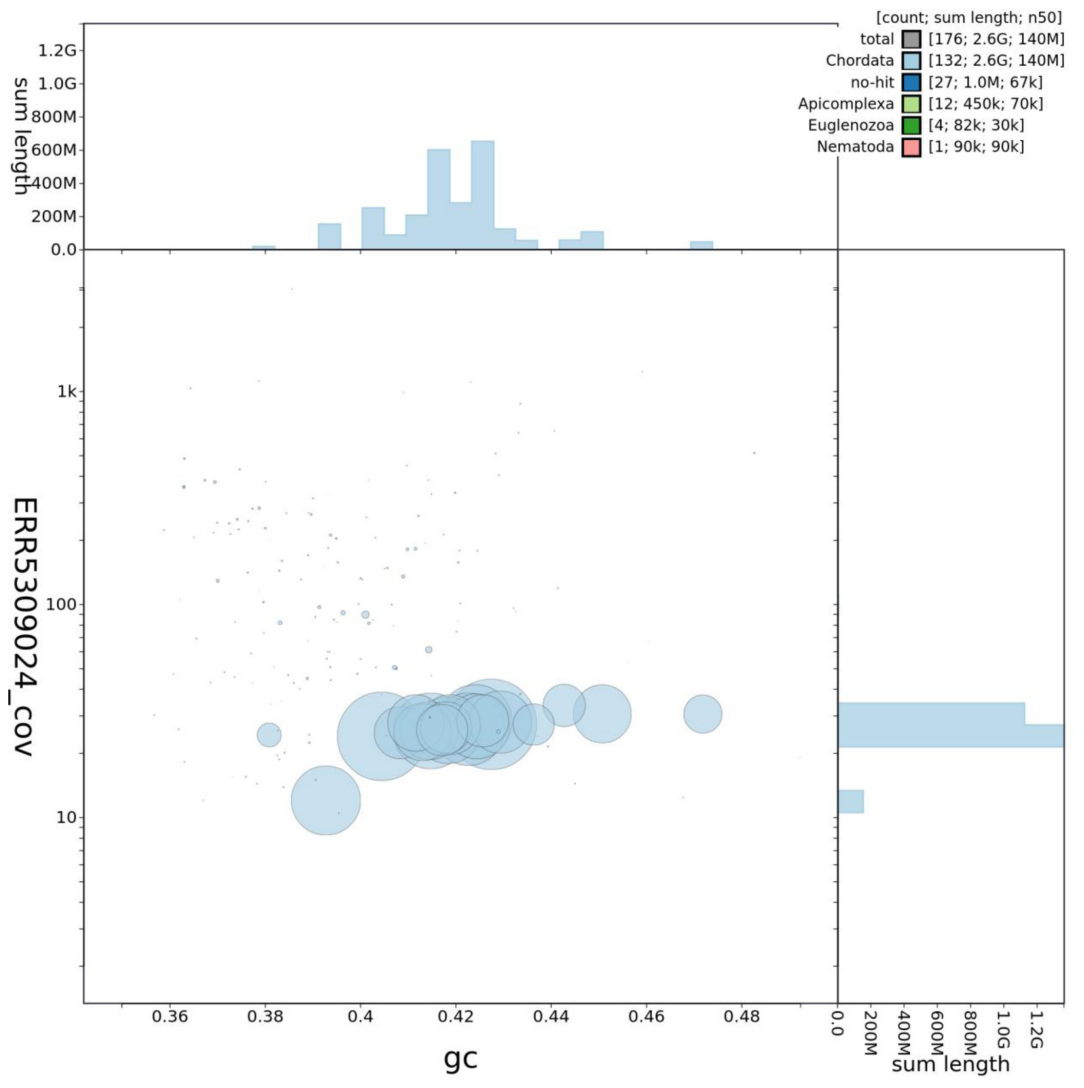
Genome assembly of *Rattus norvegicus*, mRatBN7.2: GC coverage. BlobToolKit GC-coverage plot. An interactive version of this figure is available at https://blobtoolkit.genomehubs.org/view/Rattus%20norvegicus/dataset/JACYVU01/blob?plotShape=circle.

**Figure 3.**
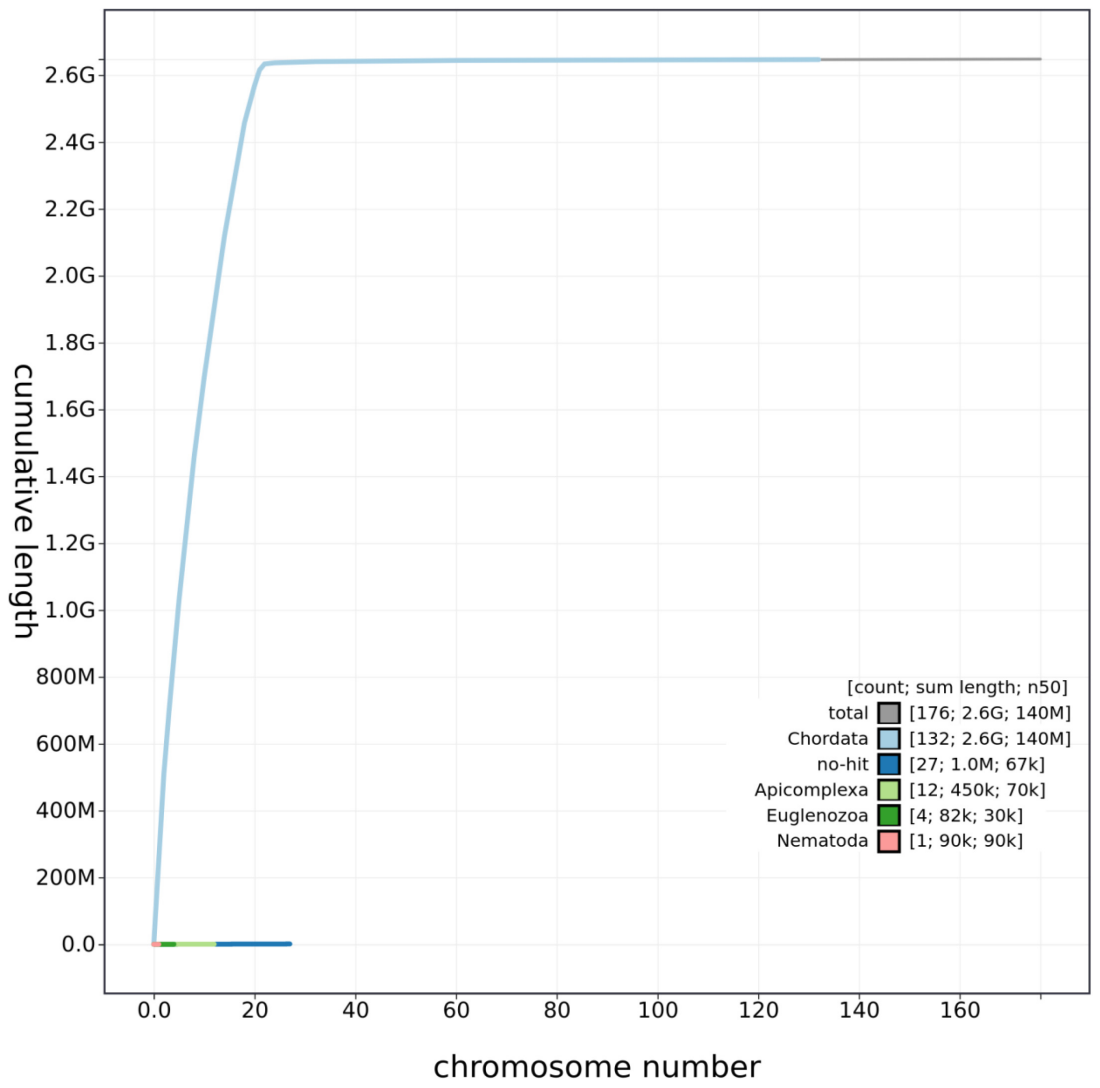
Genome assembly of *Rattus norvegicus*, mRatBN7.2: cumulative sequence. BlobToolKit cumulative sequence plot. An interactive version of this figure is available at https://blobtoolkit.genomehubs.org/view/Rattus%20norvegicus/dataset/JACYVU01/cumulative.

**Figure 4.**
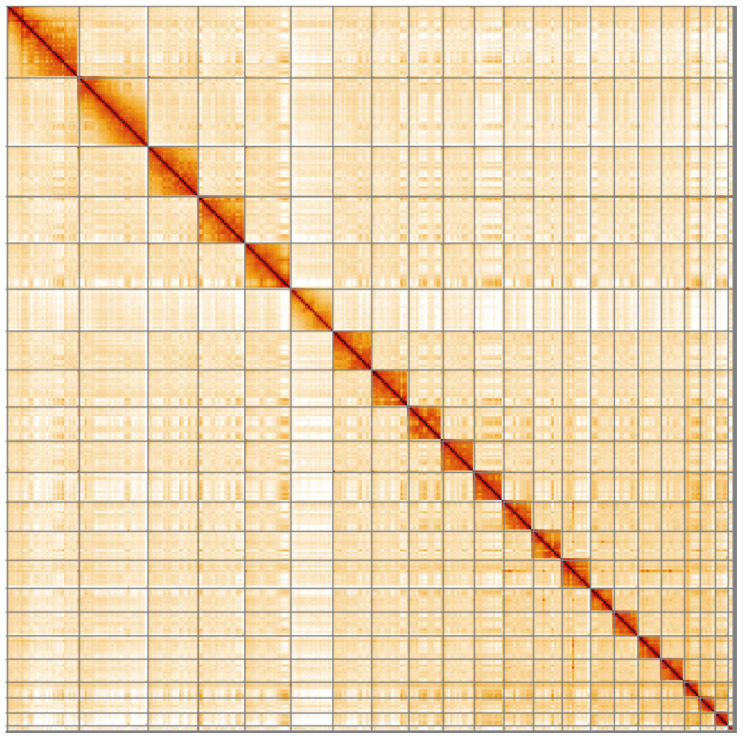
Genome assembly of *Rattus noevegicus*, mRatBN7.2: Hi-C contact map. Hi-C contact map of the mRatBN7.2 assembly, visualised in HiGlass.

**Table 2.** Chromosomal pseudomolecules in the primary genome assembly of *Rattus norvegicus* mRatBN7.2.

Accession	Chromosome	Size (Mb)	GC%
CM026974.1	1	260.52	42.8
CM026975.1	2	249.05	40.5
CM026976.1	3	169.03	42.5
CM026977.1	4	182.69	41.6
CM026978.1	5	166.88	42.3
CM026979.1	6	140.99	41.9
CM026980.1	7	135.01	42.4
CM026981.1	8	123.90	43
CM026982.1	9	114.18	41.9
CM026983.1	10	107.21	45.1
CM026984.1	11	86.24	40.8
CM026985.1	12	46.67	47.2
CM026986.1	13	106.81	41.5
CM026987.1	14	104.89	41.3
CM026988.1	15	101.77	41.2
CM026989.1	16	84.73	41.8
CM026990.1	17	86.53	42.6
CM026997.1	18	83.83	41.7
CM026992.1	19	57.34	44.3
CM026993.1	20	54.44	43.7
CM026994.1	X	152.45	39.5
CM026995.1	Y	18.32	42.2

## Methods

The Norway rat specimen (strain BN/NHsdMcwi, generation F61) was a male individual housed in a standard rodent microisolator cage at the Medical College of Wisconsin, Milwaukee, Wisconsin, USA. The animal was euthanised by CO _2_ inhalation. This procedure was approved by the Medical College of Wisconsin Institutional Animal Care and Use Committee.

DNA was extracted using an agarose plug extraction from kidney tissue following the Bionano Prep Animal Tissue DNA Isolation Soft Tissue Protocol. Pacific Biosciences CLR long read and 10X Genomics read cloud sequencing libraries were constructed according to the manufacturers’ instructions. Hi-C data were generated using the Arima v2 Hi-C kit. Sequencing was performed by the Scientific Operations DNA Pipelines at the Wellcome Sanger Institute on Pacific Biosciences SEQUEL II and Illumina HiSeq X instruments. DNA was labeled for Bionano Genomics optical mapping following the Bionano Prep Direct Label and Stain (DLS) Protocol and run on one Saphyr instrument chip flowcell.

Assembly was carried out following the Vertebrate Genome Project pipeline v1.6 ( Rhie *et al.*, 2020) with Falcon-unzip ( Chin *et al.*, 2016), haplotypic duplication was identified and removed with purge_dups ( Guan *et al.*, 2020) and a first round of scaffolding carried out with 10X Genomics read clouds using scaff10x (see Table 3 for software versions and sources). Hybrid scaffolding was performed using the BioNano DLE-1 data and BioNano Solve. Scaffolding with Hi-C data ( Rao *et al.*, 2014) was carried out with SALSA2 ( Ghurye *et al.*, 2019). The Hi-C scaffolded assembly was polished with arrow using the PacBio data, then polished with the 10X Genomics Illumina data by aligning to the assembly with longranger align, calling variants with freebayes ( Garrison & Marth, 2012) and applying homozygous non-reference edits using bcftools consensus. Two rounds of the Illumina polishing were applied. The assembly was checked for contamination and analysed using the gEVAL system ( Chow *et al.*, 2016) as described previously ( Howe *et al.*, 2021). Manual curation was performed using gEVAL, Bionano Access, HiGlass and Pretext. In addition, we used 10X *longranger* and genetic mapping data provided by LS, RWW, HC and AK to identify and resolve regions of concern. Figure 1– Figure 3 were generated using BlobToolKit ( Challis *et al.*, 2020).

**Table 3.** Software tools used.

Software tool	Version	Source
Falcon-unzip	falcon-kit 1.8.0	( Chin *et al*., 2016)
purge_dups	1.0.0	( Guan *et al*., 2020)
Bionano Solve	Solve3.4.1_09262019	https://bionanogenomics.com/downloads/bionano-solve/
SALSA2	2.1	( Ghurye *et al*., 2019)
scaff10x	4.2	https://github.com/wtsi-hpag/Scaff10X
arrow	GCpp-1.9.0	https://github.com/PacificBiosciences/GenomicConsensus
longranger align	longranger align (2.2.2)	https://support.10xgenomics.com/genome-exome/software/ pipelines/latest/advanced/other-pipelines
freebayes	v1.3.1-17-gaa2ace8	( Garrison & Marth, 2012)
bcftools consensus	1.11-88-g71d744f8	http://samtools.github.io/bcftools/bcftools.html
HiGlass	1.11.6	( Kerpedjiev *et al*., 2018)
PretextView	0.0.4	https://github.com/wtsi-hpag/PretextView
gEVAL	N/A	( Chow *et al*., 2016)
BlobToolKit	1.2	( Challis *et al*., 2020)

The mitochondrial genome was assembled as part of assembly mRatBN7.1, but was replaced with the pre-existing mitochondrial assembly MT AY172581.1, which is identical. This replacement occurred as annotation already existed for the pre-existing assembly. As such, the primary assembly is now mRatBN7.2.

## Data availability

### Underlying data

NCBI BioProject: Rattus norvegicus (Norway rat) genome assembly, mRatBN7, Accession number PRJNA662962: https://www.ncbi.nlm.nih.gov/bioproject/PRJNA662962/

NCBI Assembly: mRatBN7.2 primary assembly, Accession number GCA_015227675.2: https://www.ncbi.nlm.nih.gov/assembly/GCF_015227675.2

NCBI Assembly: mRatBN7.1 alternate haplotype, Accession number GCA_015244455.1: https://www.ncbi.nlm.nih.gov/assembly/GCA_015244455.1

The genome sequence is released openly for reuse. The *R. norvegicus* genome sequencing initiative is part of the Darwin Tree of Life (DToL) project and the Vertebrate Genome Project (VGP) ordinal references programme. All raw data and the assemblies have been deposited in INSDC databases under BioProject PRJNA662962. Raw data and assembly accession identifiers are reported in Table 1.
