# Supplementary material for: The genome sequence of the Norway rat, Rattus norvegicus Berkenhout 1769
Source: Wellcome Open Res. 2021 May 18;6:118. [Version 1] doi: 10.12688/wellcomeopenres.16854.1 (PMC8495504; doi:10.12688/wellcomeopenres.16854.1)
Supplement: Peer review history

# Peer review: Reviewer response for version 1

**DOI:** 10.21956/wellcomeopenres.18591.r46049

**Published:** 2021-10-06

**Authors:** Kim Pruitt

**Affiliations:** 1 National Center for Biotechnology Information, National Library of Medicine, National Institutes of Health, Bethesda, MD, USA

The article describes generating a new reference genome assembly for *Rattus norvegicus*. This update is a significant improvement over the previous reference assembly for this organism and will provide critical support to the rat research community. The article provides structured information on the biological sample, the sequencing and assembly methods, and database identifier citations.  

 I have two revision requests:

**1. Table 1**:

- Remove BioSample ID SAMEA5928170 from the table. It is a duplicate of SAMN16261960 which is the BioSample ID linked to the primary and alternate haplotype assemblies. 

**2. References:**

- Rhie *et al. *(2021) ^1^ is published now. Please update this reference to indicate the Nature citation and link to PubMed 33911273 (Towards Complete and Error-Free Genome Assemblies of All Vertebrate Species).
- I note there is a newer BUSCO publication ^2^, please consider if it is more relevant to cite.

Are sufficient details of methods and materials provided to allow replication by others?

Yes

Is the rationale for creating the dataset(s) clearly described?

Yes

Are the datasets clearly presented in a useable and accessible format?

Yes

Are the protocols appropriate and is the work technically sound?

Yes

Reviewer Expertise:

My area of expertise is genome annotation, gene and sequence curation, data management, and product management.

I confirm that I have read this submission and believe that I have an appropriate level of expertise to confirm that it is of an acceptable scientific standard.

## References

[1] : Towards complete and error-free genome assemblies of all vertebrate species. *Nature* .2021;592(7856) : 10.1038/s41586-021-03451-0 737-746 10.1038/s41586-021-03451-0 33911273PMC8081667

[2] : BUSCO Update: Novel and Streamlined Workflows along with Broader and Deeper Phylogenetic Coverage for Scoring of Eukaryotic, Prokaryotic, and Viral Genomes. *Mol Biol Evol* .2021;38(10) : 10.1093/molbev/msab199 4647-4654 10.1093/molbev/msab199 34320186PMC8476166

# Peer review: Reviewer response for version 1

**DOI:** 10.21956/wellcomeopenres.18591.r46050

**Published:** 2021-09-28

**Authors:** Zhihua Jiang

**Affiliations:** 1 Comparative Genome Biology Program, Department of Animal Sciences, Washington State University, Pullman, WA, USA

Howe and colleagues report an updated reference genome assembly for the Norway rat, *Rattus norvegicus*. The team extracted DNA from kidney tissue collected from a male rat (BN/NHsdMcwi), and sequenced the DNA at 80x genome coverage with PacBio long-reads and at 31x genome coverage with 10X genomics short reads, followed by chromosomal confirmation of primary assembly using Hi-C reads at 29x genome coverage. Like all “Data Notes” published in *Wellcome Open Research*, the manuscript involves four core figures reporting genome assembly: 1) metrics, 2) GC coverage, 3) cumulative sequence and 4) Hi-C contact map plus three core tables demonstrating 1) genome data accession numbers, 2) chromosomal assembly information and 3) software tools used in the study. Recently, we mapped alternative polyadenylation sites to the newest reference genome and found dramatic improvements as compared to previous versions. The advanced genome resources will certainly facilitate functional annotation of the rat genome and promote the initiation of new research fronts to understand the complicated relationships between genome and phenome for better use of the species to model health and diseases in humans.

*Genome assembly nomenclature.* Based on the NCBI collection, there are ten assemblies of the *Rattus norvegicus* species deposited there so far. As shown in Comment Table 1, each submitter was free to name their assembly. Rnor_6.0 and its previous versions have served as representative reference genomes for a while, which were, however, replaced by mRatBN7.2. As stated by the authors, a female from generation F14 contributed to Rnor_6.0, while a male from generation F61 was used to build the assembly of mRatBN7.2.  In fact, both individuals belonged to the same colony, or the BN/NHsdMcwi strain. Perhaps that is why the authors assigned the version as 7.2, rather than 1.2, for example. My guess is that mRatBN would mean something like a male (m) rat representing Brown Norway (BN). Although the genome is indeed derived from a male, its sequences of autosomes and chromosome X can be used for any female research.  As such, labeling a male-specific assembly is not necessary. In addition, the word “rat” is rather simple, because it is not specific to the *Rattus norvegicus* species. For example, the *Rattus rattus* species is the black rat, which has a nuclear genome with 18 autosomes and sex chromosomes X and Y. Therefore, I would suggest that genome assembly nomenclature be standardized for the *Rattus norvegicus* species. For example, we may use this format: Rnor_Strain (abbreviation for a strain)_xx (version number). Accordingly, mRatBN7.2 may be renamed as Rnor_BN_7.2. Hopefully, the community can discuss this further.

*Genome description consistency.* Generally speaking, assembly and annotation of a genome is an endless task as information evolves. Some inconsistencies need to be addressed or explained in order for the manuscript to be officially published. In terms of genome size, the authors stated that “The genome sequence is 2.44 gigabases in span” in the Abstract, but “a total length of 2.65 Gb” was presented in the Genome Sequence Report section. As shown in Comment Table 1, the latter claim is inconsistent with the NCBI report. In addition, the authors also need to double check the numbers of contigs, scaffolds and their N50 and L50 values as discrepancies exist between Table 1 (reported by the authors) and Comment Table 1 (collected from NCBI). Interestingly, the authors listed PRJNA662962 as the BioProject number, which is different from what is listed at NCBI (PRJNA677964). In fact, PRJNA662962 is not wrong either, but it contains four sub-projects: PRJNA662791, PRJNA663241, PRJNA677964 and PRJEB43118. Nevertheless, PRJNA677964 is directly linked to the assembly GCA_015227675.2. As listed in Table 2, the nuclear genome of the *Rattus norvegicus* species is split into 22 chromosomal pseudomolecules, including 20 autosomes and 2 sex chromosomes. As such, the claim on “20 chromosomal-level scaffolds representing 20 autosomes and the X and Y sex chromosomes” would certainly cause confusion.

*Genome report expansion?* No doubt, the current version of the manuscript strictly follows the Data Note styles so its focus is on assembly more than annotation. If possible, the team should report any changes in 1) genome structure – genes and gene-related sequences (exons, introns, UTRs and pseudogenes, for example) and intergenic DNA (genome-wide repeats and other intergenic regions) and 2) gene collection – how many genes are terminated, how many genes are renamed (based on new gene nomenclature), how many genes are overlapped and how many new genes are added to the reported assembly.

Comment Table 1. Genome assemblies deposited at NCBI for the Norway rat, *Rattus norvegicus*.

Are sufficient details of methods and materials provided to allow replication by others?

Yes

Is the rationale for creating the dataset(s) clearly described?

Yes

Are the datasets clearly presented in a useable and accessible format?

Partly

Are the protocols appropriate and is the work technically sound?

Yes

Reviewer Expertise:

Comparative Genome Biology; Genome Sequencing; Functional Analysis; Alternative Transcriptome

I confirm that I have read this submission and believe that I have an appropriate level of expertise to confirm that it is of an acceptable scientific standard.
